# An archive of longitudinal recordings of the vocalizations of adult Gombe chimpanzees

**DOI:** 10.1038/sdata.2015.27

**Published:** 2015-05-26

**Authors:** Frans X. Plooij, Hetty van de Rijt-Plooij, Martha Fischer, Michael L. Wilson, Anne Pusey

**Affiliations:** 1 International Research-institute on Infant Studies, 6814 CE Arnhem, the Netherlands; 2 Macaulay Library, Cornell Lab of Ornithology, Ithaca, NY 14850, USA; 3 Departments of Anthropology and Ecology, Evolution and Behavior, University of Minnesota, Minneapolis-St Paul, MN 55455, USA; 4 Department of Evolutionary Anthropology, Duke University, Durham, NC 27708, USA

**Keywords:** Language, Animal behaviour, Biological anthropology, Long-term memory

## Abstract

Studies of chimpanzee vocal communication provide valuable insights into the evolution of communication in complex societies, and also comparative data for understanding the evolution of human language. One particularly valuable dataset of recordings from free-living chimpanzees was collected by Frans X. Plooij and the late Hetty van de Rijt-Plooij at Gombe National Park, Tanzania (1971–73). These audio specimens, which have not yet been analysed, total over 10 h on 28 tapes, including 7 tapes focusing on adult individuals with a total of 605 recordings. In 2014 the first part of that collection of audio specimens covering the vocalizations of the immature Gombe chimpanzees was made available. The data package described here covers the vocalizations of the adult chimpanzees. We expect these recordings will prove useful for studies on topics including referential signalling and the emergence of dialects. The digitized sound recordings were stored in the Macaulay Library and the Dryad Repository. In addition, the original notes on the contexts of the calls were translated and transcribed from Dutch into English.

## Background & Summary

Chimpanzees produce a wide variety of vocalizations, ranging from barely audible grunts to loud screams and pant-hoots that can be heard at distances of 1–2 kilometres^1–3^. These vocalizations play important roles in the complex social behaviour of chimpanzees, and have attracted growing interest from researchers^4–18^. Because chimpanzees are one of the two living species most closely related to humans, researchers have been particularly interested in insights that chimpanzee vocalizations can provide to studies of language evolution^19–21^. Here we report on a dataset that will prove useful for answering various questions about chimpanzee vocal communication: recordings of adult chimpanzees made at Gombe National Park, Tanzania (1971–1973).

The late Hetty H. C. van de Rijt-Plooij and her husband Frans Plooij recorded these vocalizations and contextual information as part of their dissertation research. The calls have not yet been analysed, but have been digitized and archived at the Macaulay Library (Data Citation 1) with extensive metadata (see section ‘Data Records’) for each recording. We previously described the dataset of recordings from immature individuals^22^; here we describe the dataset of recordings from adults.

Supplementary data files are available from Dryad (Data Citation 2). All adult individuals were recorded longitudinally for nearly 2 years, just like the immature individuals. Table 1 presents the names, birth dates, age class, sex, span of longitudinal recordings in years/months, and the number of recordings in which each individual was involved. The total number of recordings is 605.

We envisage that this collection of vocalizations may be used for numerous studies including investigation of the existence of dialects, the influence of body size on sound production, and the use of vocalizations in referential signalling.

Controversy continues over whether regional variation in chimpanzee vocal production result from social learning (as in dialects in humans and songbirds^23^) or from some other factor. Mitani, who led the first study reporting chimpanzee dialects^24^, later reassessed whether such variation necessarily resulted from social learning. Instead, Mitani and colleagues^25^ argued that regional variation in acoustic structure could result from factors including habitat acoustics and body size. Subsequent studies have provided some additional support for the vocal learning hypothesis. For example, a study of two populations of unrelated chimpanzees in captivity found acoustic differences between the two populations^26^. Additionally, a study of four groups of wild chimpanzees found acoustic differences that were unrelated to genetic differences among individuals^5^. Nonetheless, all of these studies have been cross-sectional, rather than longitudinal, and thus cannot answer questions such as whether the acoustic structure of an individual’s vocalizations is fixed or flexible over time. Combined with archival recordings from the Gombe population made by other researchers (Marler 1967)^27–29^, Uhlenbroek (1991–93)^30^, and O’Bryan (2009–10)), the recordings described here will provide an unprecedented historical depth for understanding changes in acoustic structure of primate calls over time, in other words a longitudinal study of vocal change within the population. This longitudinal record provides a particularly valuable resource for understanding how chimpanzee ‘dialects’ emerge.

Body mass data are important for testing the extent to which vocalizations provide information about the caller’s body size. Recent studies of several species have found that one measure of acoustic structure, formant frequency dispersion, correlates with body mass^31–33^, but this has not yet been examined in chimpanzees. The Gombe study is unusual in that individuals were regularly weighed during this period^34^, making it possible to match acoustic features with body mass. Because body mass measurements were made from 1967–2000, these can also be taken into account in the analysis of longitudinal changes proposed above.

Furthermore, as our collection of recordings contains a large number of ‘tonal grunts’ such as the hoo-call (that is, quiet, low amplitude alert hoo), this allows for a study of the context in which these calls are used. A recent study has argued these calls represent functionally referential signals^35^. Additional information on the contexts in which these calls are given should prove valuable in interpreting their function.

## Methods

The location of the recordings is shown in Fig. 1 of ref. 22. All the recordings of adult vocalizations were made at a cleared feeding area in the Kakombe valley of Gombe National Park in the center of the range of the habituated community, where individual chimpanzees were regularly provided with bananas from metal boxes embedded in a closed trench attached to a building^1,36^. Chimpanzees frequently visited the feeding area and the recordist waited inside the building for their arrival. When chimpanzees were present in the feeding area, the recordist stood at a distance of 5–15 meters from the chimpanzees and recorded their vocalizations with a directional Sennheiser MKH 815T microphone attached to a Nagra sound recorder (full track mono, 19.05 cm/s or 7.5 inch/s) (see Fig. 2 in ref. 22). The recordist also recorded a verbal commentary before or after the vocalizations that included the names of the chimpanzees and the names of the vocalizations they produced, together with a description of the behaviour surrounding the vocalizations. Definitions of the chimpanzee behavior categories are given in Appendix A of ref. 37.

As described in reference^22^, after the sound recordings were made, analogue audio specimens were selected from the tape and coupled with metadata that consisted of the transcriptions of the verbal commentary in Dutch and a number of other pieces of information that are described under Data Records. The analogue audio specimens were created by listening to the original recordings and cutting out the stretches of tape containing chimpanzee vocalizations. The stretches of tape were glued together and stored on 28 reels totalling 10 h of chimpanzee vocalizations, where 7 reels concerned adult individuals.

In 2010 the analogue audio specimens were digitized at a resolution of 24 bits and 96 kilohertz at the Macaulay Library. In 2014 the transcriptions of the verbal commentary to the adult recordings were translated from Dutch to English. These transcriptions and associated metadata (see Data Records) were entered into a spreadsheet and then into the Macaulay Library database (Data Citation 1).

## Data Records

**Figure 1:**
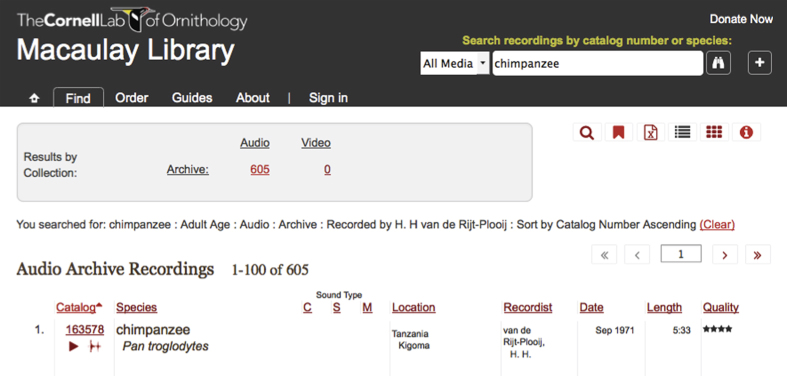
A screenshot of a Macaulay Library website search result. Clicking on the Macaulay Library Catalog number (that is 163578) will take the reader to an automatic playing of the audio along with the recording’s full set of metadata. Clicking on the red triangle plays the audio; clicking on the waveform icon brings up the audiofile in RavenViewer.

The 605 audio specimens at the Macaulay Library can be accessed directly via Data Citation 1 or by using Advanced Search, and searching for recordings with ‘Van de Rijt-Plooij, H.’ as the recordist and ‘Adult’ as Age (see Fig. 1). One can also search for vocalization types (panthoot, grunt, etc.) using the Advanced Search Notes field. As described in reference^22^ each specimen, which can be played back online, includes the following metadata: the catalog number, species name, recording date, recording geography with map, latitude/longitude, the media and equipment used, the name of the recordist, the recording length (duration), recording quality (rated according to a five star system) and notes. ‘Recording Quality’ indicates the signal-to-noise ratio with 5 stars meaning clear vocalization and very low noise in the recording. For a further specification of the measurement behind the 5 star system, see the Technical Validation section. Notes include the names of the vocalizing individual(s) together with the vocalization(s) of each individual and the behaviour and situation surrounding the vocalizations.

Many recordings contain multiple calls by multiple animals. This means the overall sample size is quite large. Table 2 (available online only) summarizes the number of each type of vocalization given by each adult individual. This table gives an indication of the frequency of the various call types and the relative contribution of each individual. It is a conservative estimate because, whenever the description gave a call type name in plural, only two calls were counted. It is striking the recordings include 303 panthoots, 141 tonal grunts and 223 grunts. These provide a robust sample size for some of the potential studies mentioned under ‘Background and Summary’.

Below, we repeat the description and use of the metadata from our previous work describing infant vocalizations^22^ with minor modifications. Metadata for all the adult individuals, cross-referenced by Macaulay Library catalog number, have been submitted to Dryad (Data Citation 2) in order to allow users to search for specific recordings beyond the capabilities currently provided by the Macaulay Library web interface. The first file of these metadata is a spreadsheet (AdultDirSounds11Dec14Final.xls) and includes the name(s) of the vocalizing individual(s), the vocalization, the behaviour, and other details. The first column of the spreadsheet contains the Macaulay Library catalog number and that is the link to the library’s database. The spreadsheet is basically the same as the Macaulay Library database except that the columns are organized in a slightly different way. From left to right the following columns can be found: ‘Macaulay catalog number’, ‘Recording Device’, ‘Focal individuals’, ‘Recordist record number’, the ‘Level of Recording’ as selected on the Nagra sound recorder, the ‘Quality outstanding’ column where an x indicates a recording that is outstanding for various reasons (such as a very clear, good-quality recording, a recording where the vocalization is without other, simultaneous vocalizations, a recording that is a good demonstration of a call type), the ‘Month’, ‘Day’ and ‘Year’ of the recording, the ‘Individuals Vocalizing’ in the recording, the ‘Individual(s) with sound/call type’, the ‘Context of vocalizations’ and behaviors surrounding the vocalizations, the ‘Macaulay Library Public Notes’ field, the ‘Microphone’, the ‘Recorder’, and the ‘Tape Speed’. As is described in the Usage Notes section, the grammar of the column containing individual(s) with sound/call type is such that the sequence of vocalizing is preserved. This gives information on who initiated calling, if several individuals called. This is important because it shows that vocalizations of others often triggered individuals to vocalize. In the column ‘Observation of the context and behaviors surrounding the vocalizations’ the presence of nearby individuals was also noted, even if they did not vocalize.

Furthermore, the Dryad data package includes the unparsed digital copies of the chimpanzee tapes (the source analog reel-to-reel media that the Macaulay Library converted to 96 kHz/24-bit files) and two additional data files. One file is the Gombe_biography (Gombe_biography-for_1971-3.xls) for the chimpanzee individuals present during the span of time that the recordings were made. The Gombe biography gives the name of the individual (column B), the estimated birth date (column C), and the sex of the individual (column I). These and other columns in the file are explained in^38^. The second file is a list of names of adult vocalizations (List of vocalizations adults.xlsx) as used in the spreadsheet (AdultDirSounds11Dec14Final.xls) and the Macaulay Library database. The first column contains the main categories of which the barks, eagle raa, grunts, hoots and screams are the most important. The second column contains the subdivisions of the barks, grunts, hoots and screams. The names in the first and the second column correspond to the call types in Table 2 (available online only). The third column contains all the word variations that were used for each main category or subdivision thereof. Before counting the frequencies of the vocalizations (given in Table 2 (available online only)), these word variations were converted into the name of the main category or subdivision.

## Technical Validation

The same validation procedures as described in our previous subadult work^22^ have been used to support the present adult audio recordings.

**Figure 2:**
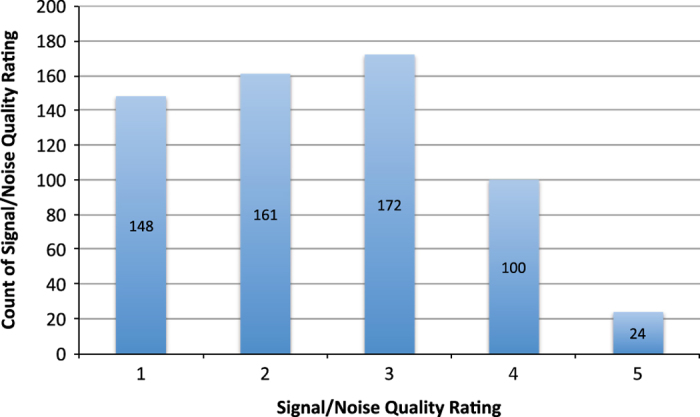
The frequency distribution of the absolute number of recordings (y-axis) over the ratio of signal power to noise power (SNR) expressed in number of stars (x-axis). The average SNR over 605 recordings is 2.49.

The ‘Quality’ of the sound recordings in the Macaulay Library is an informal and rough Indication of the ratio of signal power to noise power (SNR). Five stars means that the recording has an SNR of 50:1 (3.9% of the 605 recordings were given this rating); four stars means an SNR of roughly 40:1 (16.5%); three stars conveys an SNR of roughly 30:1 (28.5%); two stars points to an SNR of roughly 20:1 (26.6%); and one star indicates SNR of less than 10:1 (24.5%.)). The frequency distribution of the absolute number of recordings (y-axis) over the ratio of signal power to noise power (SNR) expressed in number of stars (x-axis) is given in Fig. 2. It is striking that the modus is 3 (as compared to 2 in the corresponding figure 4 of the subadult work), while the frequency of SNR=1 is higher for the adults as compared with the subadults. Consequently, the average SNR is the same for adults and subadults.

We were not able to conduct inter-observer reliability tests, because nearly all recordings were taken by one person: Hetty van de Rijt-Plooij. In our previous article^22^ on immature audio recordings, we describe an intra-observer reliability test conducted on videotape of infant chimps. This video presented many challenges for scoring: the focal infant was playing with another infant, a few other individuals were present, and many interactions occurred in a short period of time. The results of the test were satisfactory and only minor/subtle mistakes were made. The conditions under which the recordings of adults were made presented fewer such challenges, and we are confident that the recordist accurately identified the individual calling and other key information recorded for each call.

## Usage Notes

‘Individual(s) with sound/call type’ (Column K of the metadata spreadsheet ‘AdultDirSounds 11Dec14Final.xls’) gives the names of all vocalizing individuals together with the vocalization(s) they produce. A note of ‘uncertain’ behind a name means the recordist is not quite sure the vocalization came from that individual; ‘UN’ means ‘unknown individual(s)’; ‘GEN’ means ‘General’ or ‘the whole group’; ALL means all individuals present; HUM means ‘human’; BAB means ‘baboon’. The names plus vocalization are separated by ‘,‘ (comma). This column makes ‘cross-references’ superfluous. The Grammar of column K is as follows:

- A comma followed by a single space separates vocalizations following each other immediately, or between the last vocalization of one individual and the name of the next individual in the sequence. All the vocalizations between two names belong to the individual of the first name.
- ‘…’ indicates that some time passes by between one vocalization and the next.
- Parenthical comments, such as ‘(huu)’, which is a Dutch dipthong, ‘(hoo)’, ‘(soft)’ or other remarks after the name of the vocalization describes how the vocalization sounds or gives a qualification or a general remark concerning the sound or the recording process. Whenever it says: ‘ recording needle trembling’, the literal translation of the original note would be ‘recording knob shaking’. However, because we do not understand how such a knob can shake, it is translated instead as ‘needle trembling’.
- ‘General’ means: the whole group.

In Column L (‘Context of vocalizations’) of the metadata spreadsheet ‘AdultDirSounds11Dec14Final.xls’ a more general behavioural context is given of the vocalizations involved in the recording. Whenever numbers are used, these refer to the distance categories as defined on page 24 of ref. 37. Each number concerns the distance of the individual having the number to the one other individual in the group having no number.

## Additional information

Table 2 is only available in the online version of this paper.

**How to cite this article:** Plooij, F. X. *et al.* An archive of longitudinal recordings of the vocalizations of adult Gombe chimpanzees. *Sci. Data* 2:150027 doi: 10.1038/sdata.2015.27 (2015).

## Supplementary Material



## Figures and Tables

**Table 1:** The names, birth dates, age class, sex, span of longitudinal recordings in years/months, and the number of recordings for each chimpanzee individual recorded in Gombe National Park in the period 1971–73

**Animal Name (abbreviation)**	**Birth Date (approximately)**	**Age Class**	**Sex**	**Age span longitudinal recordings (years/months)**	**Number of Recordings**
FLO	02-jul-19	Adult	F	52–54 years	7
HUGO (HG)	02-jul-36	Adult	M	35–37 years	19
GOLIATH (GOL)	02-jul-37	Adult	M	34–36 years	21
MIKE (MK)	02-jul-38	Adult	M	33–35 years	24
HUGH (HH)	02-jul-44	Adult	M	27–29 years	2
MADAM B (MB)	02-jul-45	Adult	F	26–28 years	2
HUMPHREY (HM)	02-jul-46	Adult	M	25–27 years	58
FABEN (FB)	02-jul-47	Adult	M	24–26 years	45
DE	02-jul-48	Adult	M	23–25 years	4
WILLY WALLY (WW)	02-jul-49	Adult	M	22–24 years	3
MELISSA (ML)	02-jul-49	Adult	F	22–24 years	35
PASSION (PS)	02-jul-49	Adult	F	22–24 years	9
NOPE (NP)	02-jul-50	Adult	F	21–23 years	5
CHARLIE (CH)	02-jul-51	Adult	M	20–22 years	29
EVERED (EV)	02-jul-52	Adult	M	19–21 years	48
PALLAS (PL)	02-jul-52	Adult	F	19–21 years	54
ATHENA (AT)	02-jul-52	Adult	F	19–21 years	11
FIGAN (FG)	02-jul-53	Adult	M	18–20 years	73
GODI (GI)	02-jul-53	Adult	M	18–20 years	15
NOVA (NV)	02-jul-53	Adult	F	18–20 years	32
GIGI (GG)	02-jul-54	Adult	F	17–19 years	14
SATAN (ST)	02-jul-55	Adult	M	16–18 years	17
MIFF (MF)	02-jul-56	Adult	F	15–17 years	7
JOMEO (JJ)	02-jul-56	Adult	M	15–17 years	29
FIFI (FF)	02-jul-58	Adult	F	13–15 years	24
WINKLE (WK)	02-jul-58	Adult	F	13–15 years	68
SPARROW (SW)	02-jul-58	Adult	F	13–15 years	16
SNIFF (SF)	02-jul-59	Adolescent	M	12–14 years	3
GILKA (GK)	02-jul-60	Adolescent	F	11–13 years	1
SHERRY (SH)	02-jul-61	Adolescent	M	10–12 years	8
FLINT (FT)	01-mrt-64	Juvenile	M	7.2–9.0 years	6
GOBLIN (GB)	06-sep-64	Juvenile	M	6.7–8.5 years	8
MUSTARD (MU)	22-nov-65	Juvenile	M	5.5–7.3 years	1
ATLAS (AL)	25-sep-67	Infant	M	43–65 months	2
MOEZA (MZ)	20-jan-69	Infant	F	28–50 months	1
SKOSHA (SS)	27-mrt-70	Infant	F	14–30 months	4
PLATO (PT)	07-sep-70	Infant	M	10–29 months	6
GREMLIN (GM)	19-nov-70	Infant	F	7–27 months	2
FREUD (FD)	22-mei-71	Infant	M	0–20 months	13
PROF (PF)	26-okt-71	Infant	M	0–15 months	1
Span of longitudinal recordings in months are only given for the infants. The total number of recordings is 727.					

**Table 2:** Counts of call types by adult individuals

	**Individual ID**																													
**Call type**	**AT**	**CH**	**DE**	**EV**	**FB**	**FF**	**FG**	**FLO**	**GEN**	**GG**	**GI**	**GOL**	**HG**	**HH**	**HM**	**JJ**	**MB**	**MF**	**MK**	**ML**	**NP**	**NV**	**PL**	**PS**	**ST**	**SW**	**UN**	**WK**	**WW**	**TOTAL**
**banging**	0	0	0	0	0	0	0	0	1	0	0	0	0	0	2	0	0	0	0	0	0	0	0	0	0	1	0	0	0	4
**bark**	4	0	1	3	3	1	3	0	2	6	3	2	0	0	0	0	0	1	2	0	2	2	0	1	1	6	15	8	1	67
**beep**	0	0	0	0	0	0	1	0	0	0	0	0	0	0	0	0	0	0	0	0	0	0	0	0	0	0	0	0	0	1
**burp**	0	0	0	0	0	0	0	0	0	0	0	0	0	0	0	0	0	0	0	0	0	1	0	0	0	0	0	0	0	1
**cough**	0	0	0	1	0	0	0	0	0	0	0	0	0	0	4	0	0	0	13	1	0	0	6	0	1	2	0	2	0	30
**coughing**	0	0	0	0	0	0	0	0	0	0	0	1	0	0	0	0	0	0	0	0	0	0	0	0	0	0	0	0	0	1
**crying**	0	0	0	0	0	0	2	0	0	0	0	0	0	0	0	0	0	0	0	0	0	0	1	0	0	0	5	0	0	8
**drumming**	0	1	0	1	2	0	5	0	0	0	0	1	0	0	1	5	0	0	0	0	0	0	0	0	2	0	2	0	0	20
**eagle raa**	1	0	0	0	4	0	0	0	0	0	0	0	0	0	0	0	0	0	0	0	0	0	0	0	0	0	13	0	0	18
**effort grunt**	0	0	0	0	0	0	0	0	0	0	0	0	0	0	0	0	0	0	0	0	0	0	0	0	0	0	0	0	0	0
**excitement**	0	0	0	0	0	0	0	0	11	0	0	0	0	0	0	0	0	0	0	0	0	0	0	0	0	0	1	0	0	12
**foodbark**	0	0	0	0	1	0	1	0	13	0	1	3	3	0	3	0	0	0	0	0	0	0	0	0	0	0	13	0	0	38
**groan**	0	0	0	0	1	0	0	0	0	0	0	0	0	0	1	0	0	0	0	0	0	0	0	0	0	0	0	0	0	2
**grunt**	1	6	0	8	23	5	34	3	4	4	1	5	2	0	11	7	0	4	7	19	0	16	24	1	5	0	9	24	0	223
**hoot**	3	2	0	2	9	4	60	0	4	0	1	3	4	3	4	8	0	0	2	0	0	1	0	0	3	0	5	7	0	125
**laugh**	0	0	0	0	0	4	2	0	0	0	0	6	0	0	0	0	0	1	0	0	0	0	0	0	4	0	0	0	0	17
**lipsmacking**	0	0	0	0	0	0	0	0	0	0	0	0	0	0	0	0	0	0	0	0	0	0	0	0	0	0	3	0	0	3
**pantbark**	0	0	0	4	3	0	0	0	3	0	1	0	0	0	0	0	0	1	1	0	1	0	0	1	0	1	2	1	0	19
**pantgrunt**	0	0	0	2	2	0	0	2	1	0	1	1	0	0	0	4	1	4	0	0	0	1	5	2	0	0	5	1	0	32
**panthoot**	2	15	2	23	8	9	37	1	39	1	10	9	3	0	36	17	0	0	5	9	2	3	1	1	7	2	45	16	0	303
**panting**	2	0	0	1	2	2	3	0	2	0	0	0	2	0	1	0	1	0	1	0	1	1	2	0	2	0	0	0	0	23
**rough grunt**	5	1	2	6	2	2	3	0	0	5	1	0	0	0	0	0	0	2	2	7	2	2	11	1	0	0	5	6	1	66
**scream**	4	0	1	1	6	5	6	0	4	3	1	1	1	0	2	0	0	2	2	1	5	2	4	0	2	5	18	7	2	85
**sigh**	0	1	0	0	0	0	0	2	0	1	0	0	0	0	0	0	0	0	0	0	0	1	0	0	0	0	0	0	0	5
**silence**	0	0	0	0	0	1	0	0	4	0	0	0	0	0	0	0	0	2	0	0	1	0	0	0	3	0	10	4	0	25
**slap**	0	0	0	0	0	0	1	0	0	0	0	0	0	0	0	0	0	0	0	0	0	0	0	0	0	0	0	0	0	1
**slapstamp**	0	0	0	0	0	0	1	0	0	0	0	0	0	0	0	0	0	0	0	0	0	0	0	0	0	0	0	0	0	1
**sneeze**	0	0	0	0	1	0	0	0	0	1	0	0	0	0	0	0	0	0	0	0	0	0	0	0	0	0	0	0	0	2
**sound**	1	0	0	0	1	0	0	0	0	0	1	1	0	0	0	0	0	0	0	1	0	0	0	0	0	3	10	0	0	18
**squeak**	0	2	0	1	1	1	4	0	2	5	0	2	0	0	5	0	0	0	0	2	1	6	1	0	0	5	1	8	1	48
**squeal**	3	0	0	0	0	0	0	0	0	2	0	0	0	0	0	0	0	0	0	0	1	0	0	0	0	0	1	2	0	9
**stamp**	0	0	0	0	0	0	0	0	0	0	1	0	0	0	0	1	0	0	0	0	0	0	0	0	0	1	0	0	0	3
**teethclapping**	0	0	0	0	0	0	0	0	0	0	0	0	1	0	0	0	0	0	1	0	0	0	0	0	1	0	3	0	0	6
**tonal grunt**	5	7	0	6	14	0	6	0	2	2	0	2	8	0	6	0	0	0	6	1	4	7	14	3	3	9	9	27	0	141
**uh**	0	0	0	0	0	0	0	0	0	0	0	0	0	0	0	0	0	0	0	0	0	1	3	0	0	0	2	0	0	6
**unknown**	0	0	0	0	2	0	0	0	0	0	0	0	0	0	0	0	0	0	0	0	0	0	0	0	0	0	0	0	0	2
**vocalizations**	0	0	0	0	0	0	0	0	0	0	0	0	0	0	0	0	0	0	0	0	0	0	0	0	0	0	0	0	0	0
**waabark**	0	0	0	1	2	6	2	0	4	2	0	0	1	0	0	1	0	0	1	0	0	0	0	0	0	1	10	4	0	35
**whimper**	0	0	0	0	0	0	1	0	0	0	0	0	0	0	0	0	1	0	0	0	2	2	0	0	0	3	0	1	0	10
**whimper ho**	0	0	0	0	0	0	0	0	0	0	0	0	0	0	0	0	0	0	0	2	0	3	0	2	0	2	1	4	0	14
**yawn**	0	0	0	0	0	0	0	0	0	0	0	0	1	0	0	0	0	0	0	0	0	0	0	0	0	0	0	0	0	1
**TOTAL**	31	35	6	60	87	40	172	8	96	32	22	37	26	3	76	43	3	17	43	43	22	49	72	12	34	41	188	122	5	1425
